# Supplementary material for: Targeting alveolar epithelial cells with lipid micelle-encapsulated necroptosis inhibitors to alleviate acute lung injury
Source: Commun Biol. 2025 Apr 6;8:573. doi: 10.1038/s42003-025-08010-1 (PMC11972349; doi:10.1038/s42003-025-08010-1)

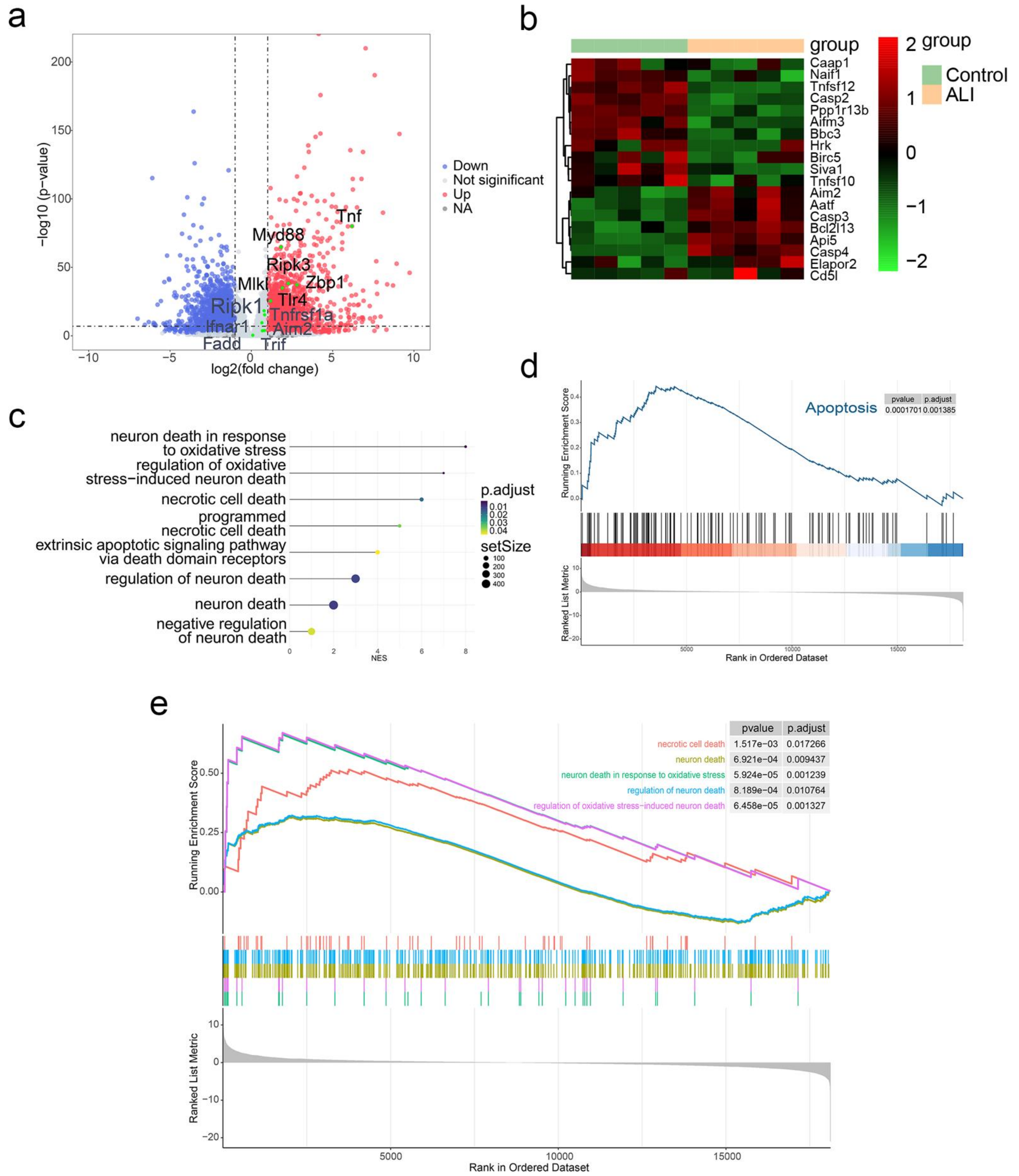

Supplementary Figure 1. Analysis of lung tissue sequencing results in control and ALI mice. (a) Volcano plots of Bulk RNA-sequence results revealed that the expression of genes involved in the necroptosis pathway was altered in ALI group. Gene names highlighted in black represent those with a p-value < 0.05 and a log2FoldChange > 1, while gene names in grey represent those with a p-value < 0.05 and a log2FoldChange < 1. (b) Heat mapping of Bulk RNA-sequence results revealed that the expression of genes involved in the apoptosis pathway was altered in ALI group. (c) A Lollipop plot was created using Bulk RNA-seq data and GO enrichment analyses. Specifically, the functional set related to death were extracted and displayed. (d) GSEA analysis using Bulk RNA-sequence data demonstrated that the apoptosis pathway was significantly activated in the lung tissue of mice in ALI group (p < 0.05). (e) A GSEA map was generated using Bulk RNA-seq data and GO-based GSEA enrichment analysis. There were the top five functional sets associated with death for presentation.

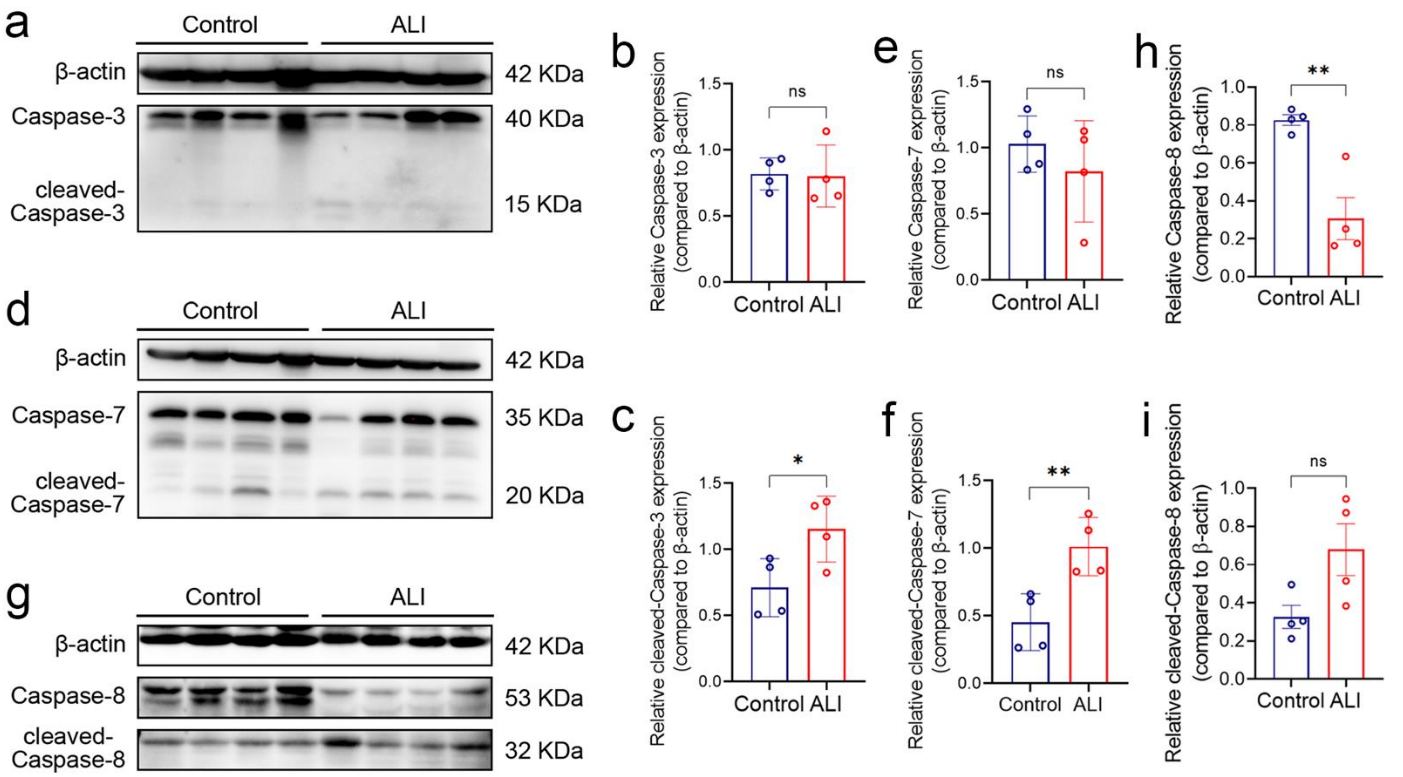

Supplementary Figure 2. Increased cleaved-Caspase-3 and cleaved-Caspase-7 in lung tissues of ALI, while decreased in Caspase-8. (a, d, g) Validation of Caspase-3 , cleaved-Caspase-3, Caspase-7 , cleaved-Caspase-7, Caspase-8 and cleaved-Caspase-8 expression in lung tissue using western blotting. (b, c, e, f, h, i) Quantitative statistical analysis of the western blotting results was performed by measuring the grayscale values of the bands using Image J, with the target bands normalized to the internal reference (β-actin). Significance was determined with p-values < 0.05, 0.01, by *, **, respectively, ns means not significant. The error bars represent the standard deviation (SD).

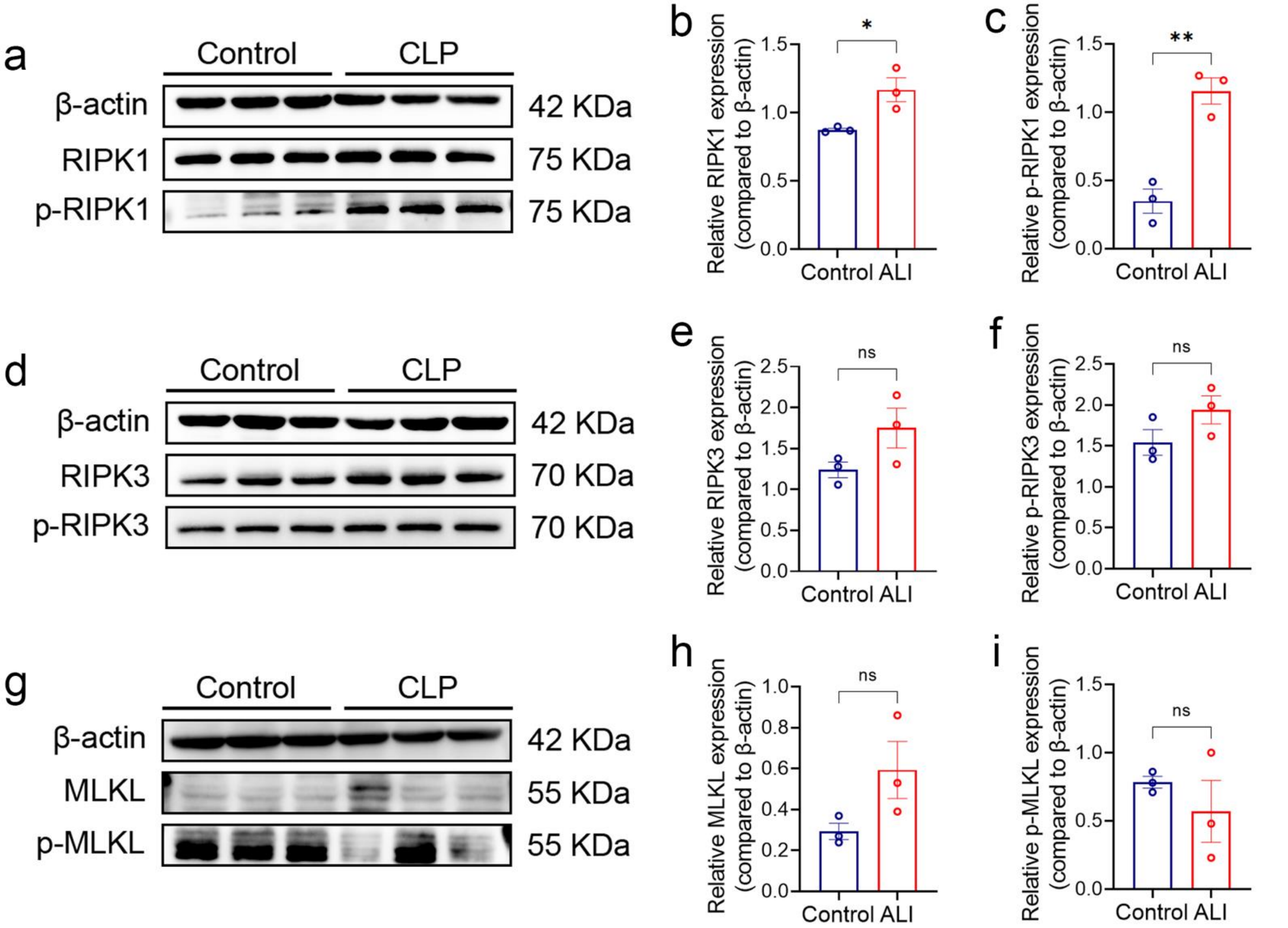

Supplementary Figure 3. Western blot results of lung tissue from mice with CLP. (a, d, g) Validation of RIPK1, p-RIPK1, RIPK3, p-RIPK3, MLKL, and p-MLKL in mouse lung tissue with CLP using western blotting. (b, c, e, f, h, i) Quantitative statistical analysis of the western blotting results was performed by measuring the grayscale values of the bands using Image J, with the target bands normalized to the internal reference (β-actin). Significance was determined with p-values < 0.05, 0.01, by *, **, respectively, ns means not significant. The error bars represent the standard deviation (SD).

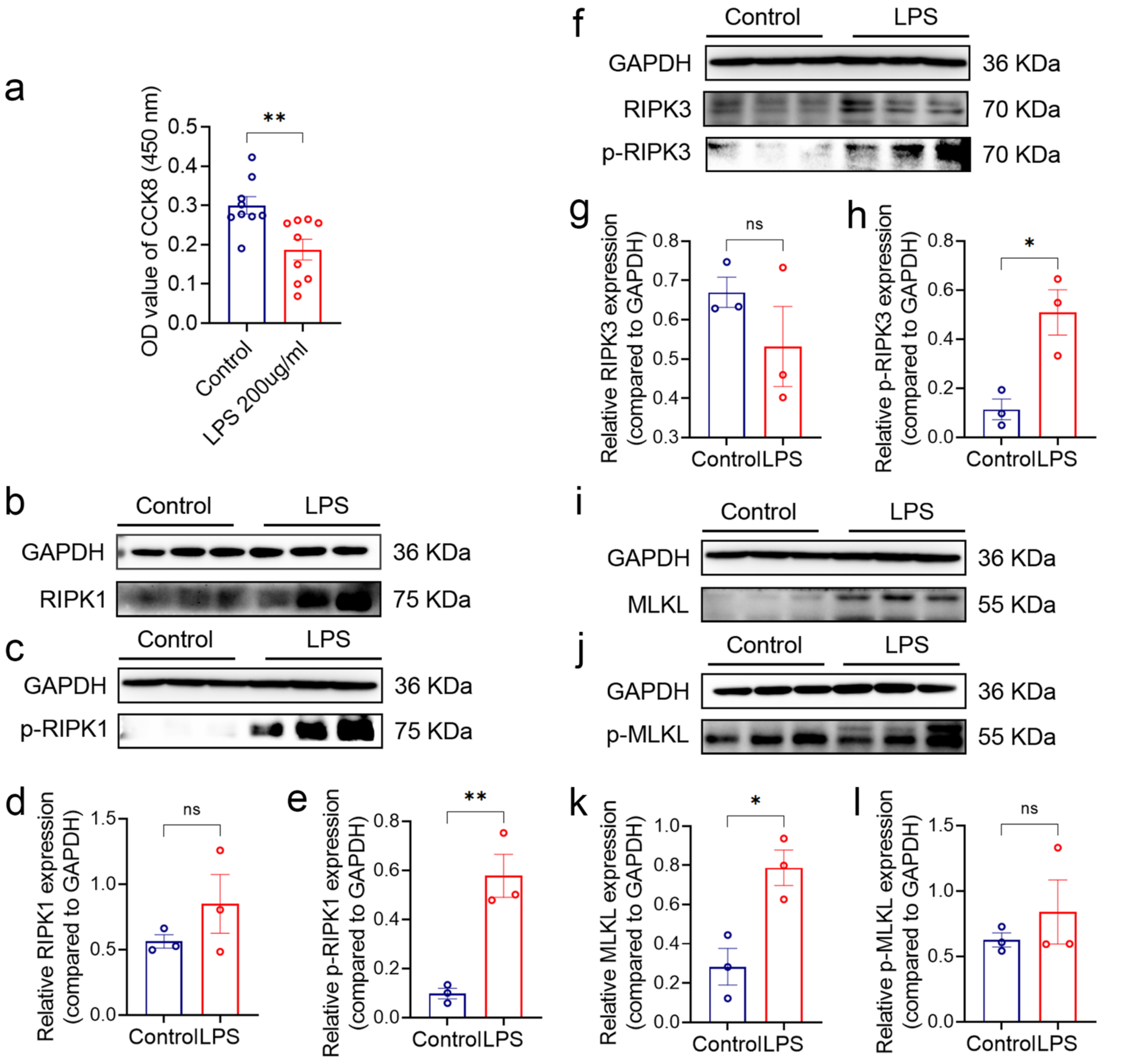

Supplementary Figure 4. RIPK1, RIPK3 and MLKL expression in LPS treated HBE cells in 24 hours in vitro. (a) Viability using CCK-8 detection of HBE after LPS treatment. (b, c, f, i, j) Western blotting was performed to verify the expression of RIPK1, p-RIPK1, RIPK3, p-RIPK3, MLKL, and p-MLKL in HBE treated with 200 μg/ml of LPS for 24 hours. The bands for p-RIPK1 and MLKL were obtained from the same membrane after multiple exposures, so the internal reference band used for the p-RIPK1 and MLKL bands is the same. (d, e, g, h, k, l) Quantitative analysis of the western blotting results was conducted by measuring the grayscale values of the bands using Image J. The target bands were normalized to the internal reference (β-actin). Significance was determined with p-values < 0.05, 0.01, by *, **, respectively, ns means not significant. The error bars represent the standard deviation (SD).

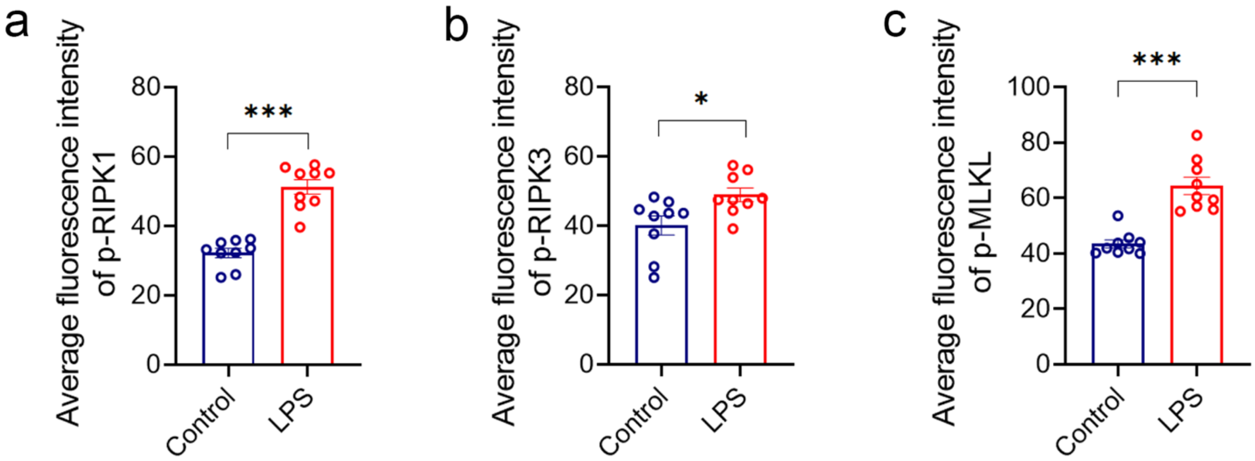

Supplementary Figure 5. Fluorescence quantification of p-RIPK1, p-RIPK3, and p-MLKL after LPS intervention in HBE cells. (a-c) Bar plots represent the average fluorescence intensity of the three fluorescent proteins. Three samples were used for each group in the experiment, and three random fields were selected for each sample. The average fluorescence intensity was calculated by using Image J. Significance was determined with p-values < 0.05, 0.001, by *, ***, respectively, ns means not significant. The error bars represent the standard deviation (SD).

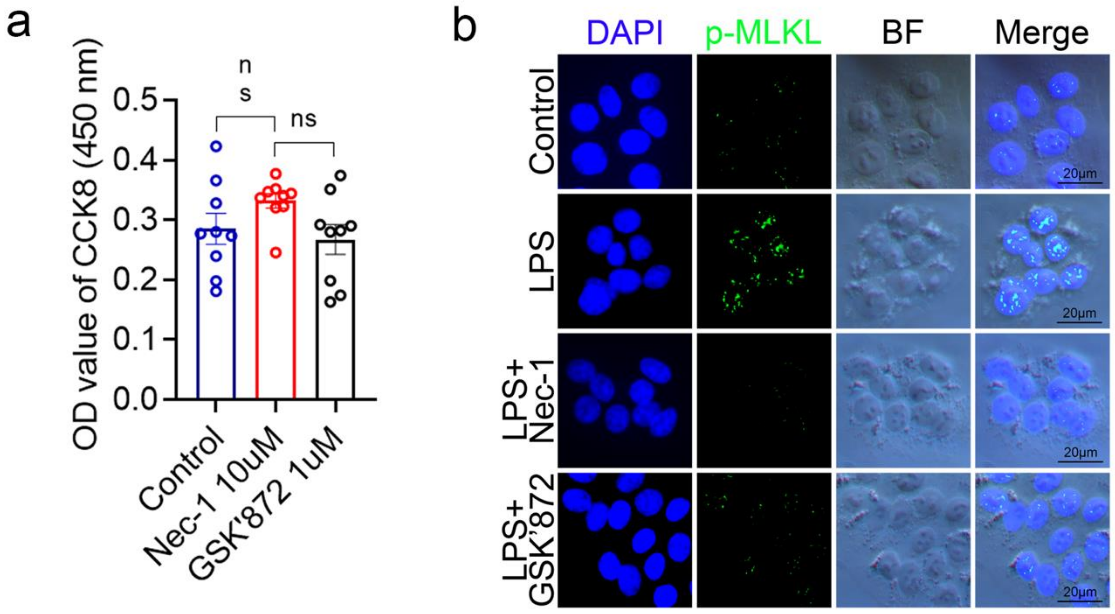

Supplementary Figure 6. GSK'872 and Nec-1 significantly reduced p-MLKL levels in HBE. (a) The effect of Nec-1 and GSK'872 on HBE viability detected with CCK-8. (b) Fluorescence microscope images were captured for HBE treated with anti-p-MLKL (green) and DAPI (blue) in the control, LPS, LPS+Nec-1 and LPS+GSK'872 treated cells. Scale markers of 20 μm were included in the reference. The error bars represent the standard deviation (SD).

a

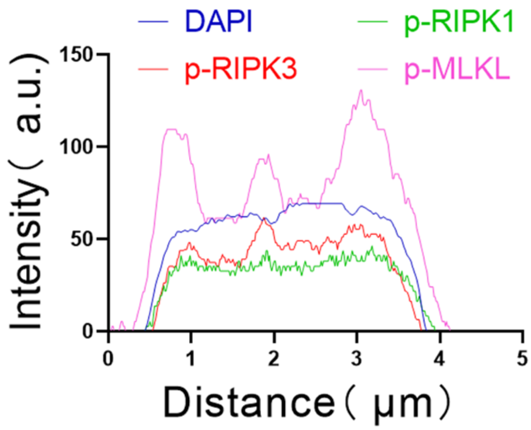

b

Cluster Scores

Cluster	Members	Respresentative	Weighted Score
0	49	Center	-686.5
		Lowest Energy	-965.8
1	30	Center	-690.1
		Lowest Energy	-785.1
2	27	Center	-740.2
		Lowest Energy	-800.6
3	26	Center	-660.8
		Lowest Energy	-728.7
4	25	Center	-809.7
		Lowest Energy	-809.7
5	25	Center	-660.8
		Lowest Energy	-794.0
6	24	Center	-674.8
		Lowest Energy	-754.8
7	24	Center	-778.8
		Lowest Energy	-778.8
8	23	Center	-746.6
		Lowest Energy	-746.6
9	22	Center	-698.4
		Lowest Energy	-720.1

Supplementary Figure 7. p-RIPK1, p-RIPK3 and p-MLKL can be present as complexes. (a) Fluorescence intensity of HBE intervened with LPS were processed with p-RIPK1 (green), p-RIPK3 (red), p-MLKL (pink), and DAPI (blue) as markers. The fluorescence intensity was quantified using image J. Consistent fluorescence peaks were observed for p-RIPK1 (green), p-RIPK3 (red), and p-MLKL (pink). (b) The binding energies required for the formation of RIPK1 and RIPK3-MLKL complexes were determined using the first 10 binding modes provided by the ClusPro website.

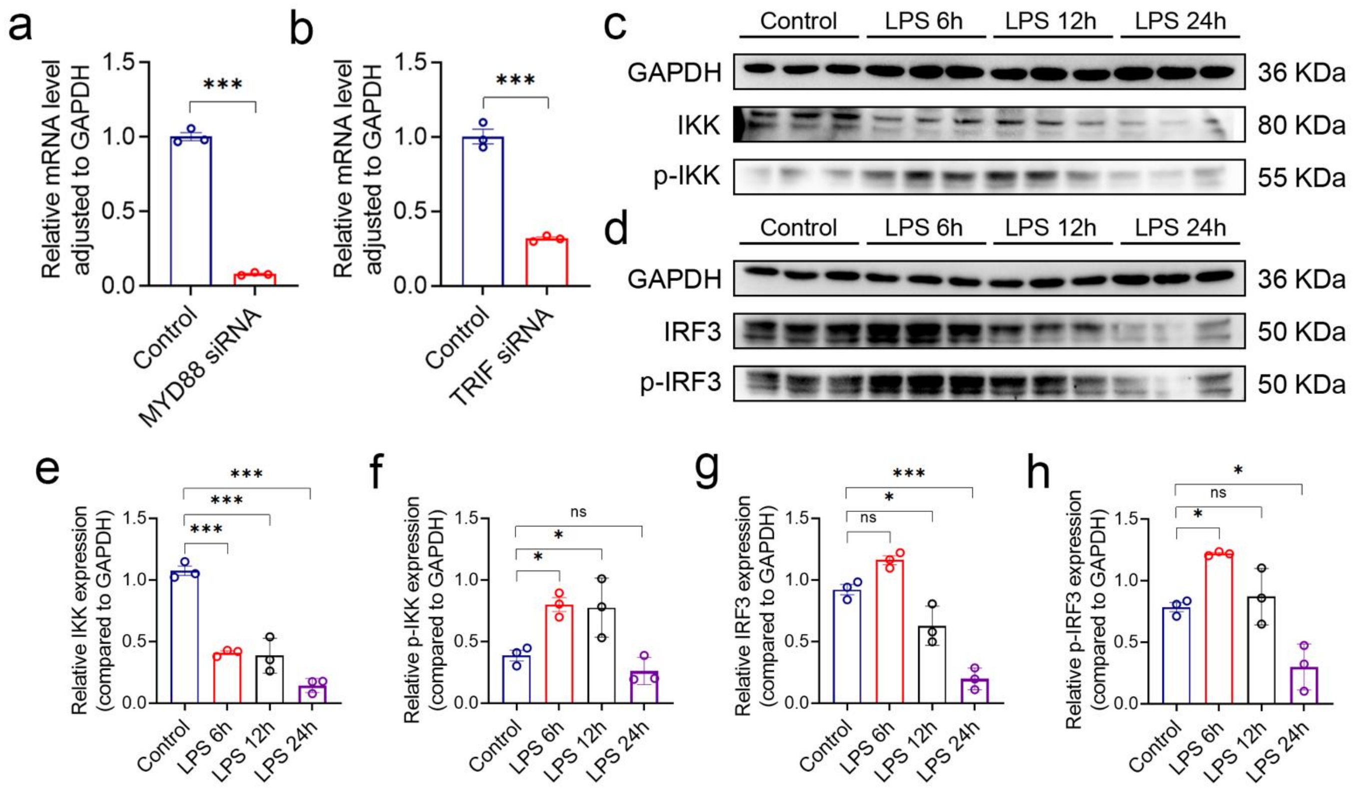

Supplementary Figure 8. The knockdown efficiency of siRNA in HBE cells and the expression levels of p-IKK and p-IRF3. (a, b) After intervening with *Myd88* siRNA and *Trif* siRNA in HBE , PCR was performed on the cell samples. The y-axis represents the relative RNA expression levels normalized to GAPDH, while the x-axis denotes the different experimental groups. Western blotting was performed to verify the expression of IKK, p-IKK (c), IRF3, and p-IRF3 (d) in HBE treated with LPS at a concentration of 200 μg/ml for 6, 12, and 24 hours, respectively. ns: no significant; *P<0.05, **P<0.01; ***P<0.001. Quantitative statistical analysis of western blotting results for the expression levels of IKK (e), p-IKK (f), IRF3 (g), and p-IRF-3 (h) in HBE following LPS treatment at various time points. The error bars represent the standard deviation (SD).

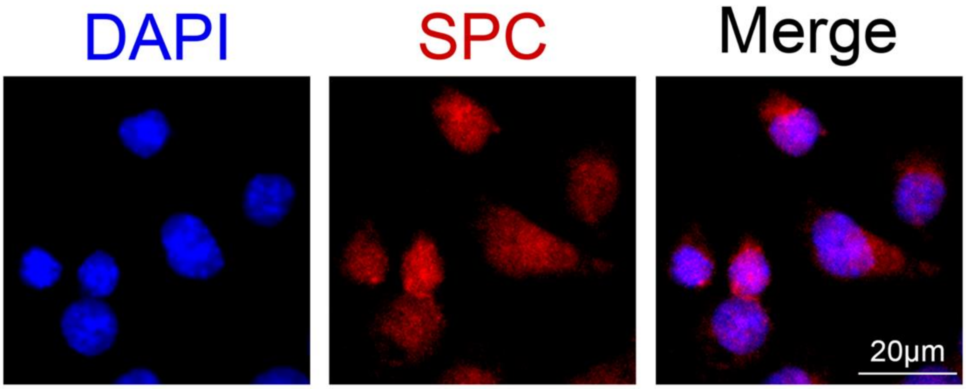

Supplementary Figure 9. MLE expresses SPC protein on the cell membrane surface. Immunofluorescence staining of MLE was performed using SPC antibody, with red indicating the expression of SPC protein in the cells and blue representing the cell nucleus. The scale bar is 20 μm.

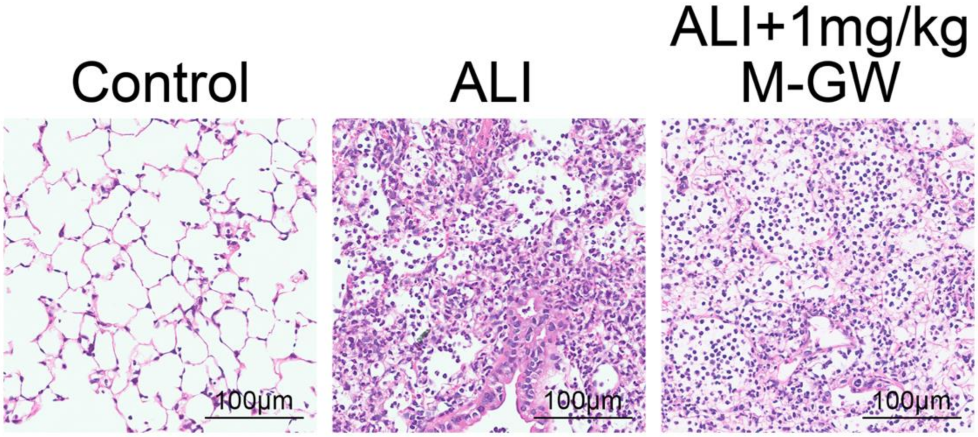

Supplementary Figure 10. The 1 mg/kg dose of M-GW did not show significant improvement in the exudation of lung tissue in ALI mice. Microscope images of HE-stained lung tissues of mice of ALI and ALI/M-GW. The scale bar is 100 μm.

Figure 2

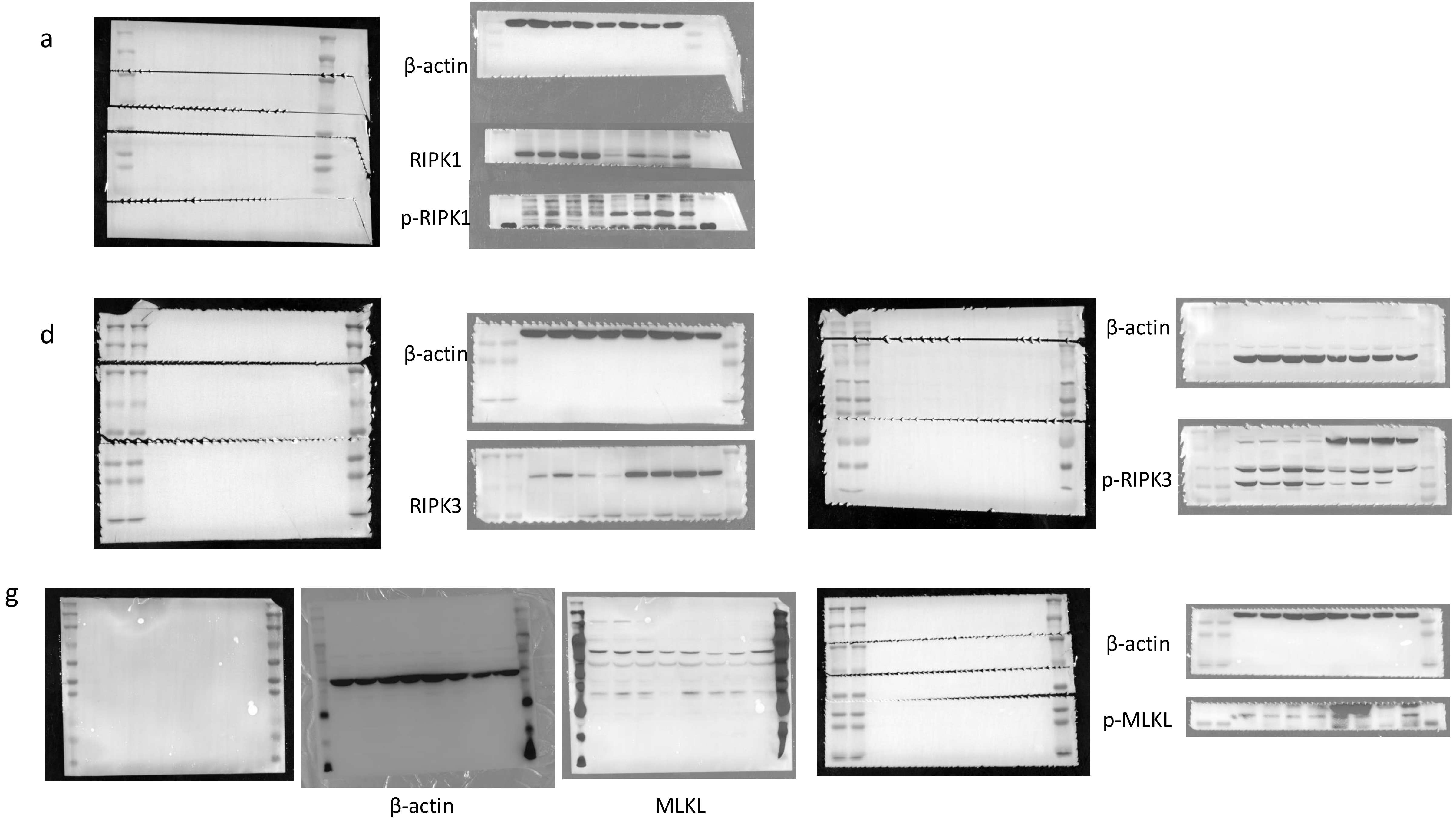
a
β-actin
RIPK1
p-RIPK1
d
β-actin
RIPK3
β-actin
p-RIPK3
g
β-actin
MLKL
β-actin
p-MLKL

Figure 3

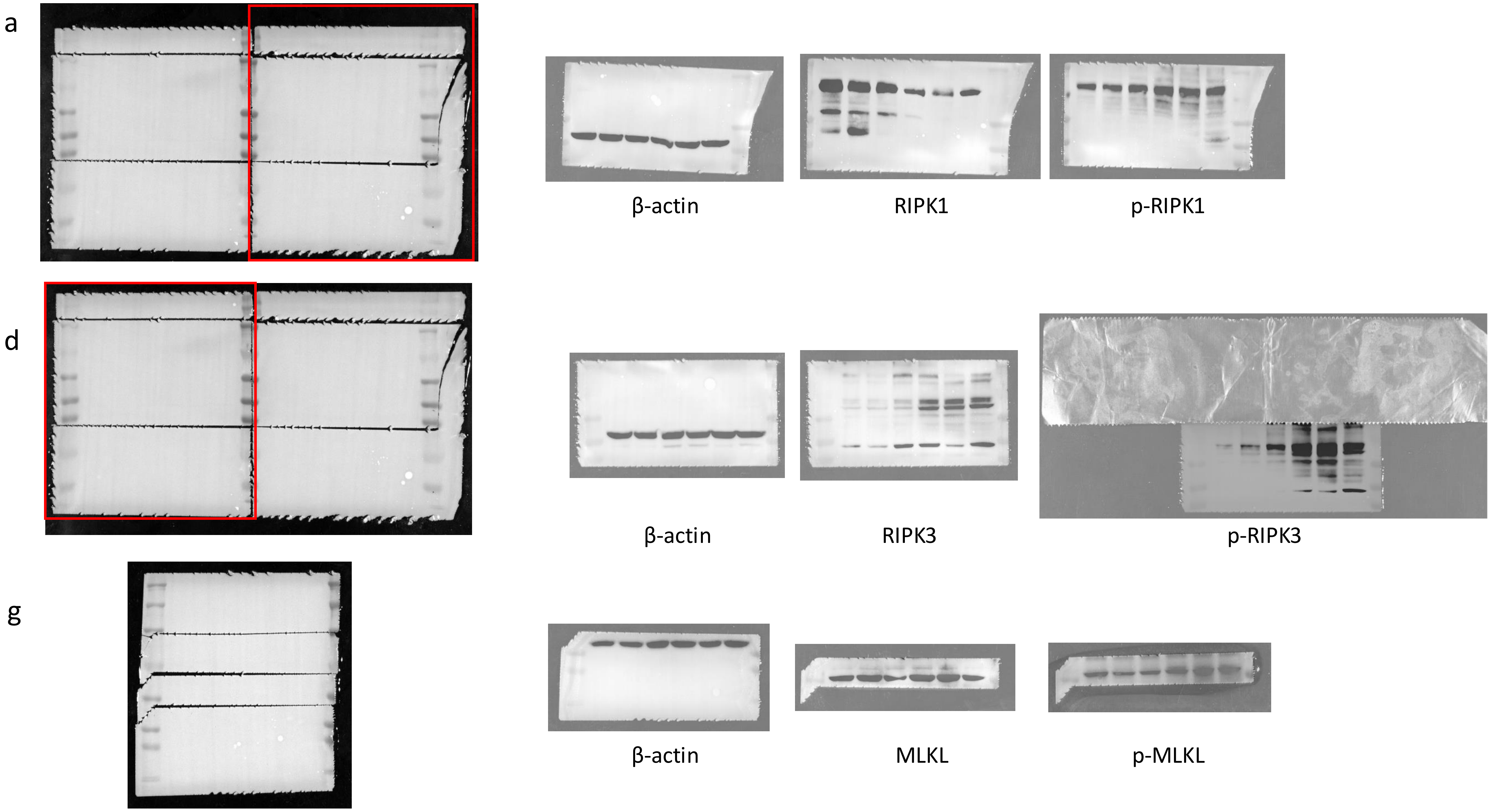

a
β-actin
RIPK1
p-RIPK1
d
β-actin
RIPK3
p-RIPK3
g
β-actin
MLKL
p-MLKL

Figure 4

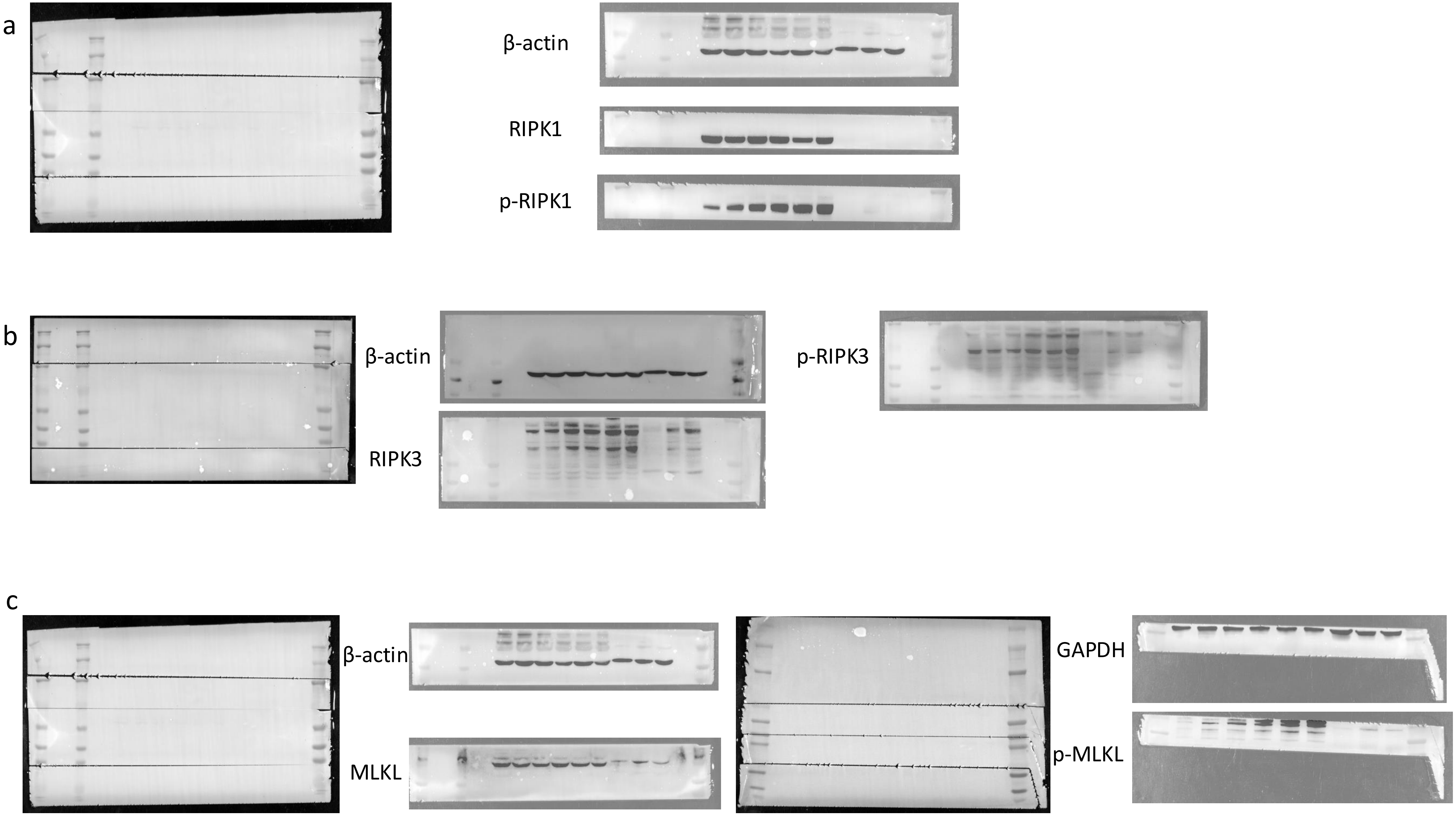
a
β-actin
RIPK1
p-RIPK1
b
β-actin
RIPK3
p-RIPK3
c
β-actin
MLKL
GAPDH
p-MLKL

Figure 4

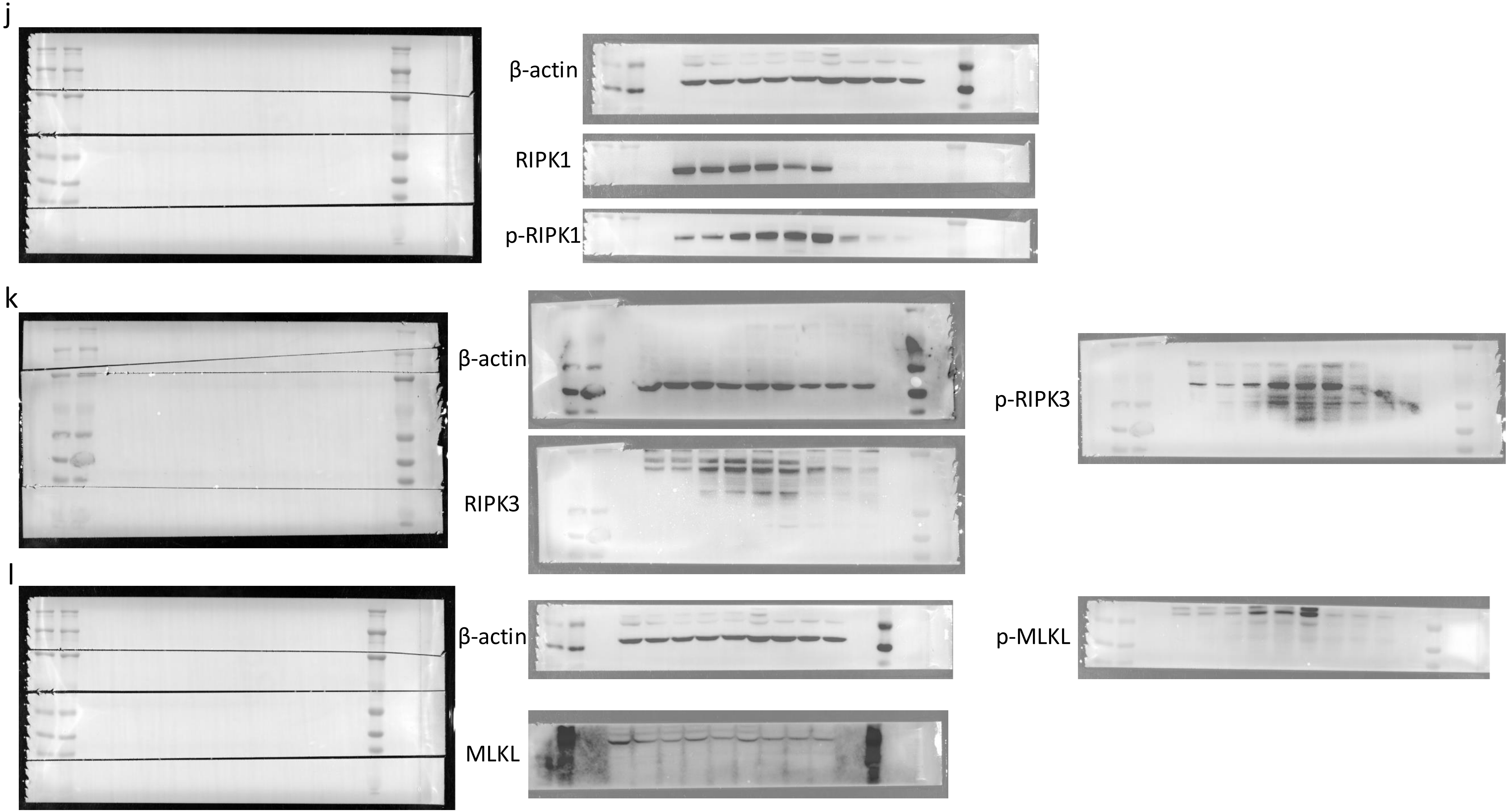
j
β-actin
RIPK1
p-RIPK1
k
β-actin
RIPK3
p-RIPK3
l
β-actin
MLKL
p-MLKL

Figure 5

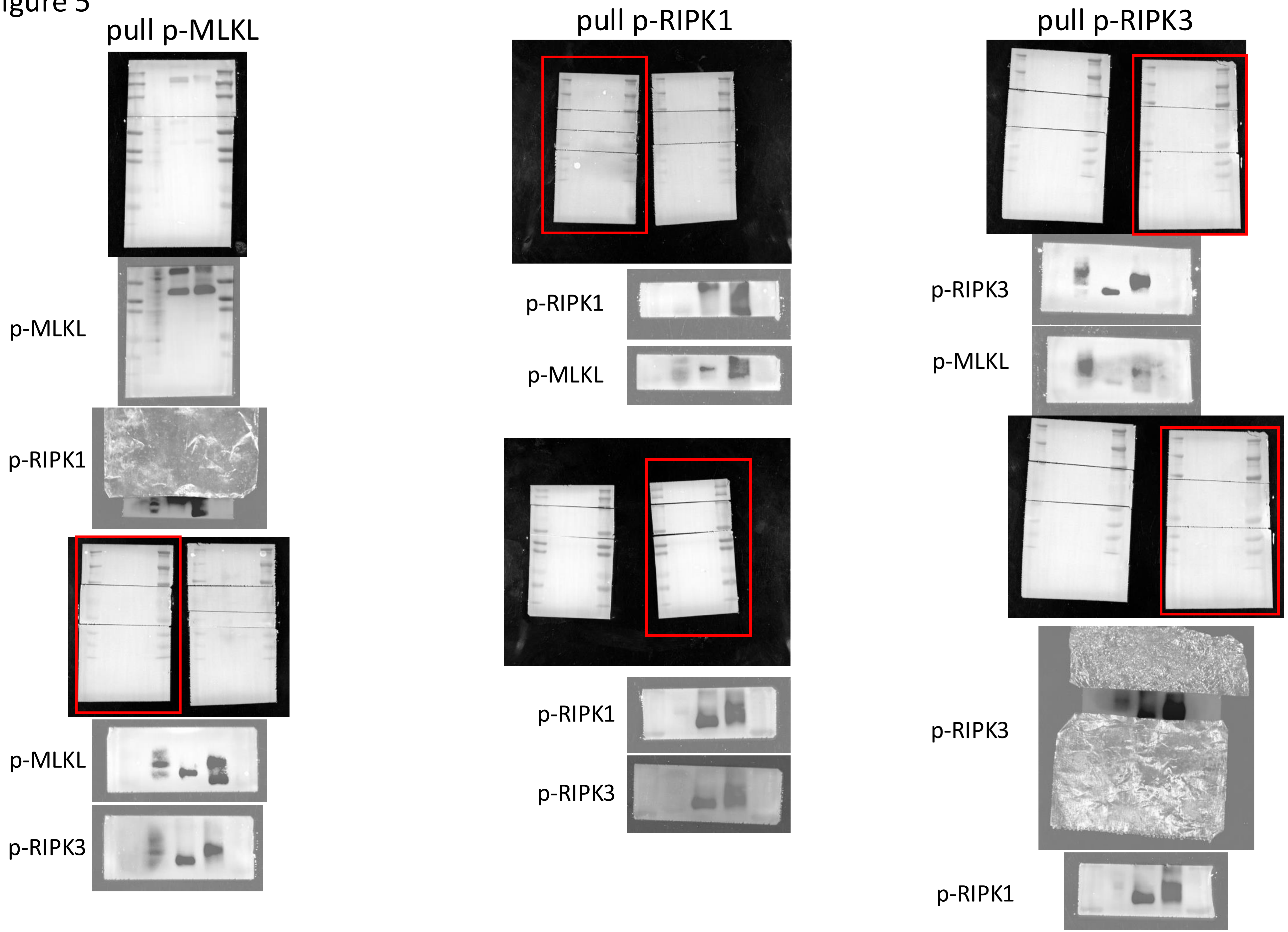
pull p-MLKL
p-MLKL
p-RIPK1
p-MLKL
p-RIPK3
pull p-RIPK1
p-RIPK1
p-MLKL
p-RIPK1
p-RIPK3
pull p-RIPK3
p-RIPK3
p-MLKL
p-RIPK3
p-RIPK1

Figure 6

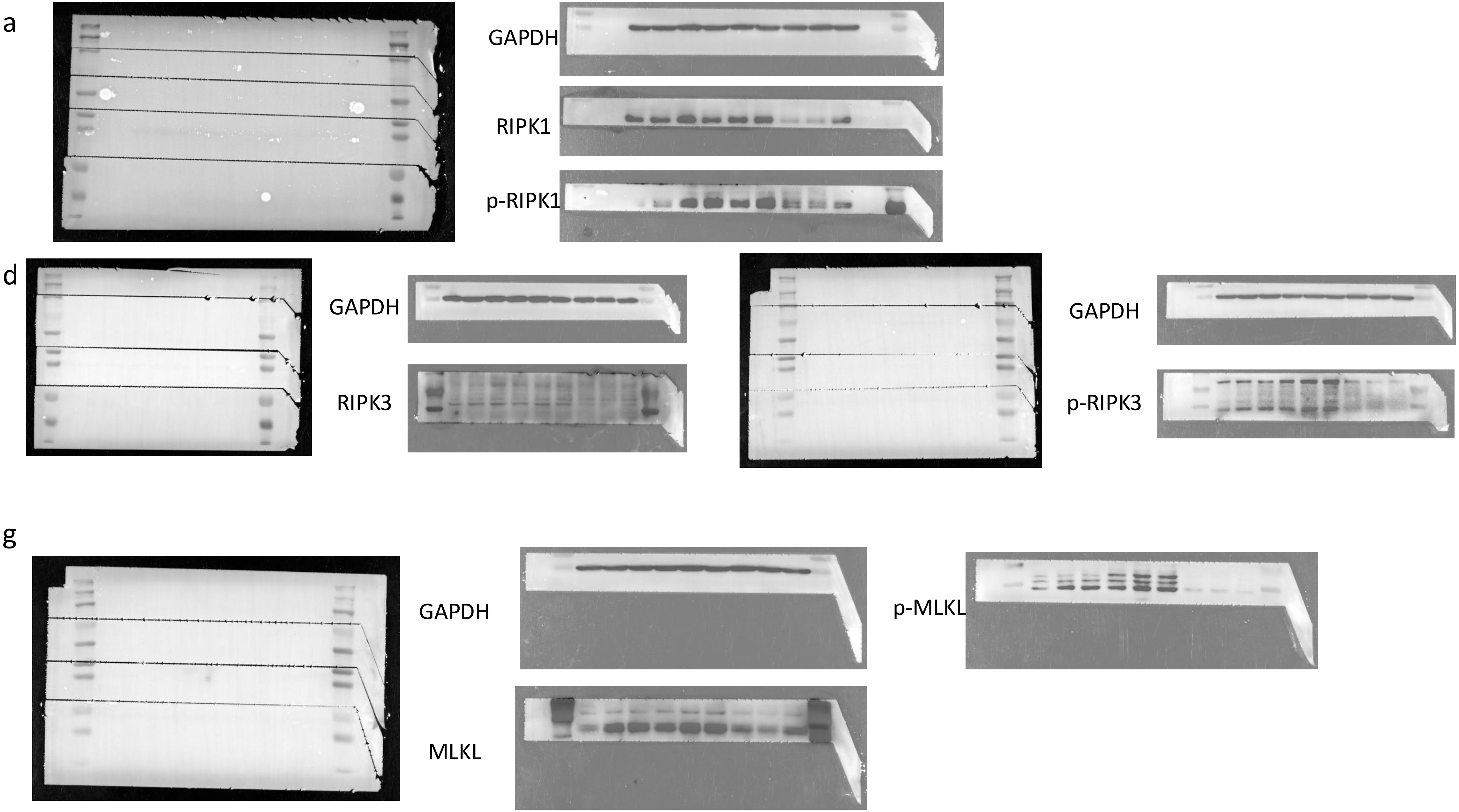
a
GAPDH
RIPK1
p-RIPK1
d
GAPDH
RIPK3
GAPDH
p-RIPK3
g
GAPDH
p-MLKL
MLKL

Figure 6

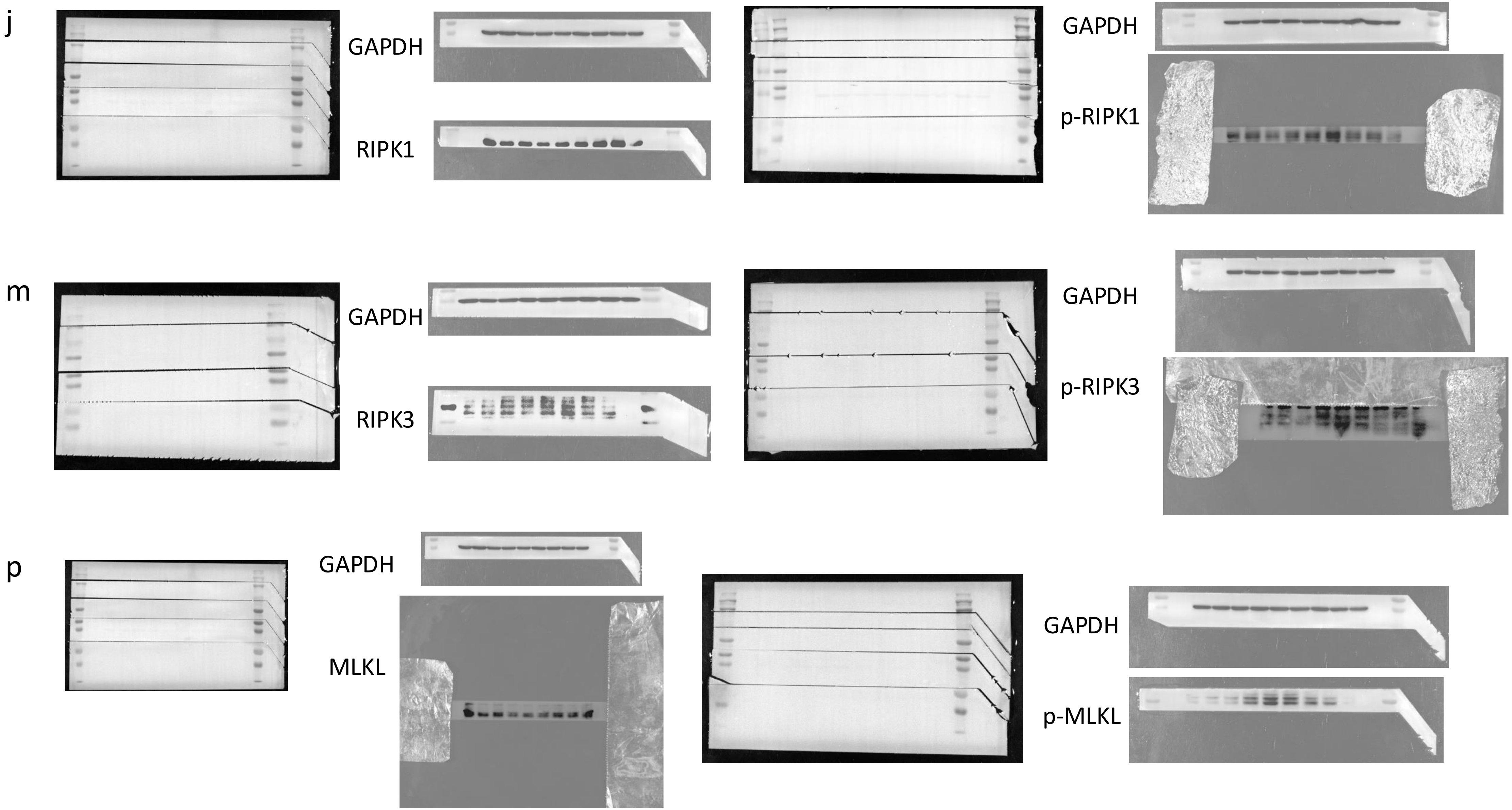
j
GAPDH
RIPK1
GAPDH
p-RIPK1
m
GAPDH
RIPK3
GAPDH
p-RIPK3
p
GAPDH
MLKL
GAPDH
p-MLKL

Figure S2

a

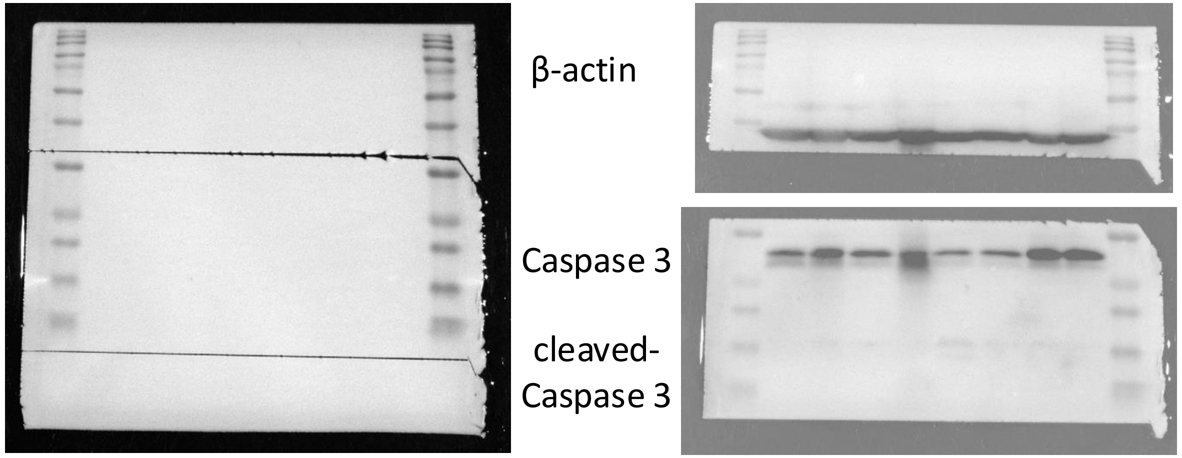

d

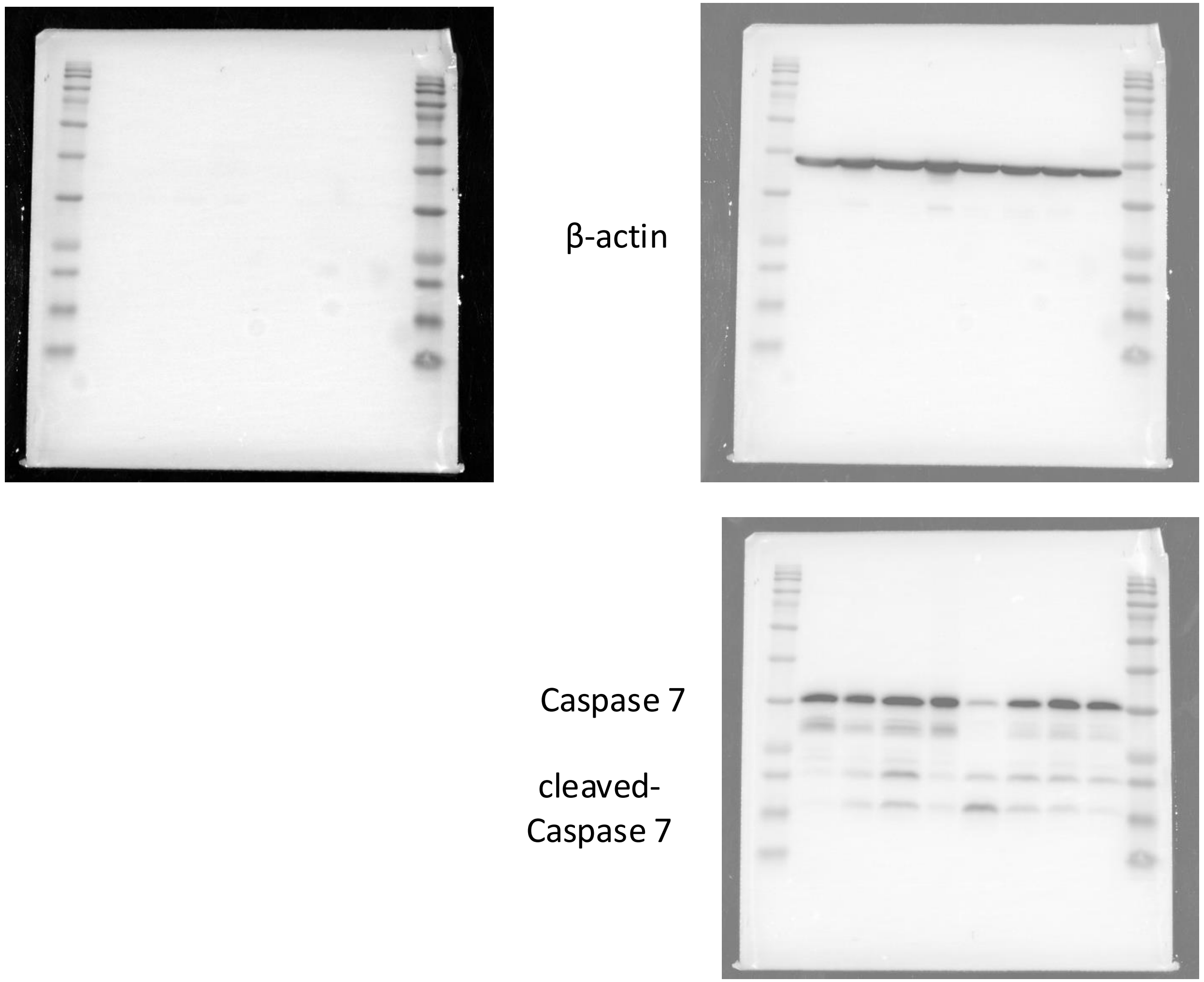

g

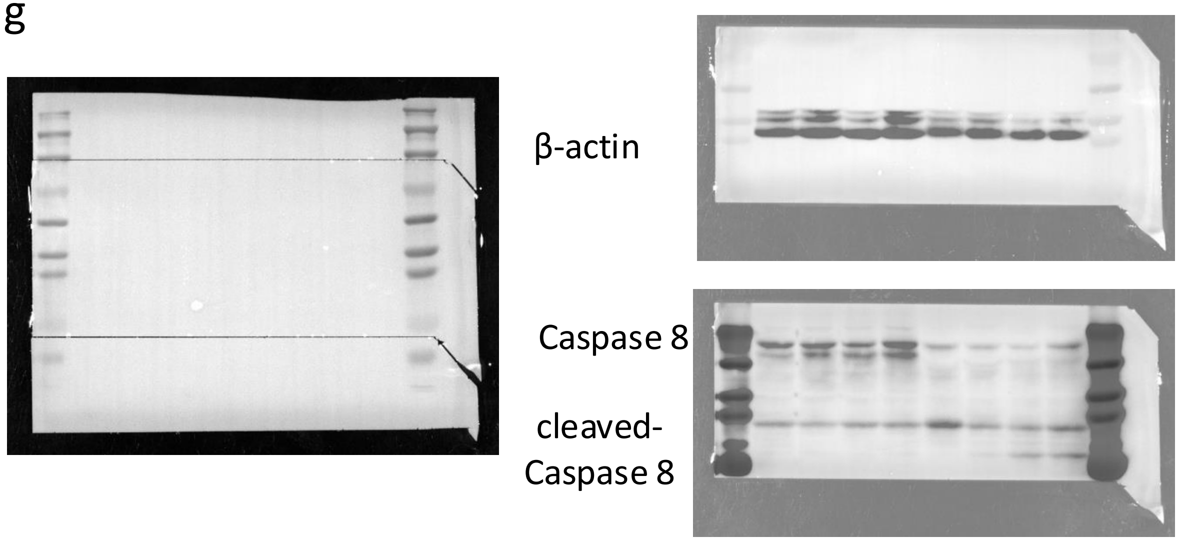

Figure S3

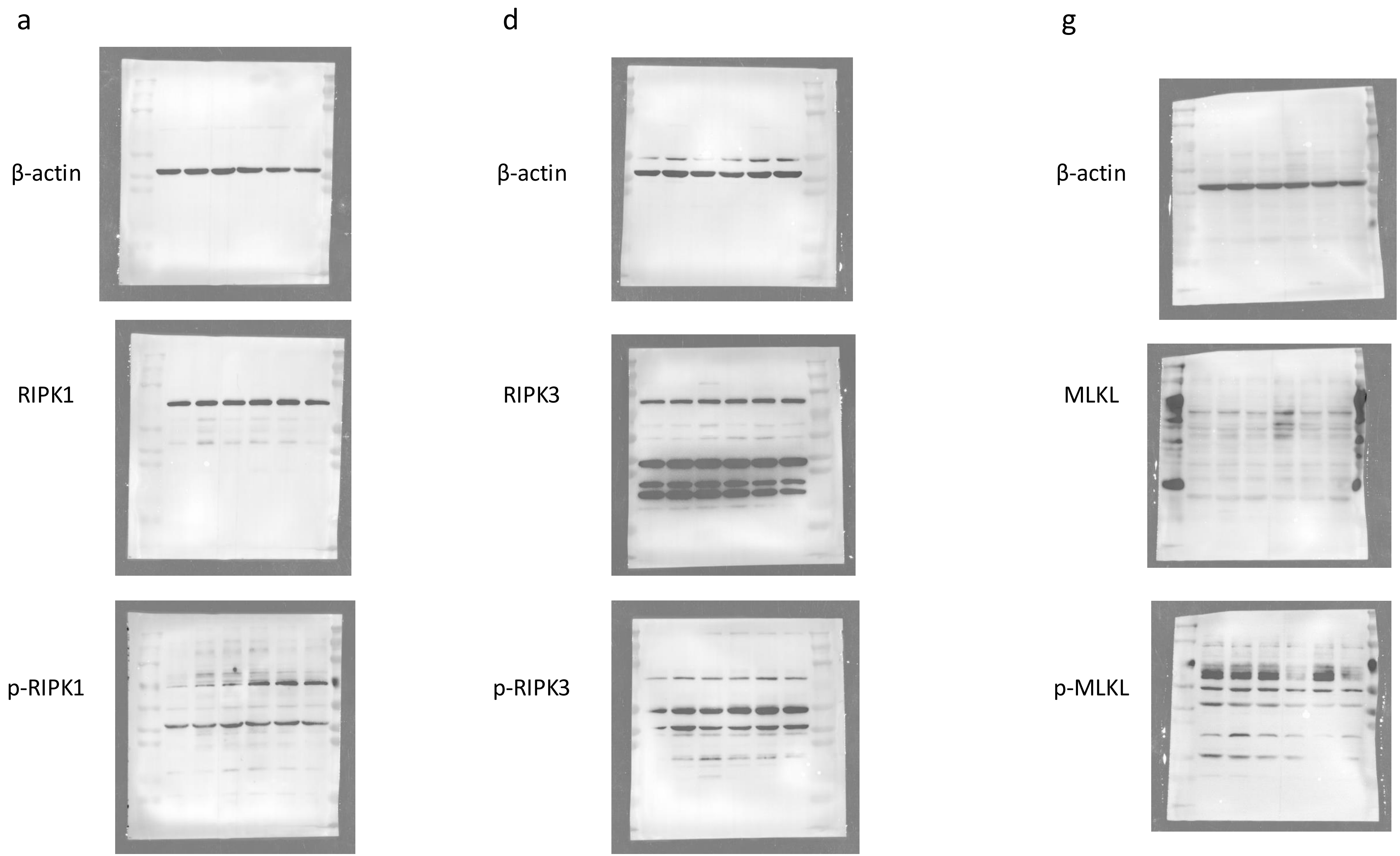
a
β-actin
RIPK1
p-RIPK1
d
β-actin
RIPK3
p-RIPK3
g
β-actin
MLKL
p-MLKL

Figure S4

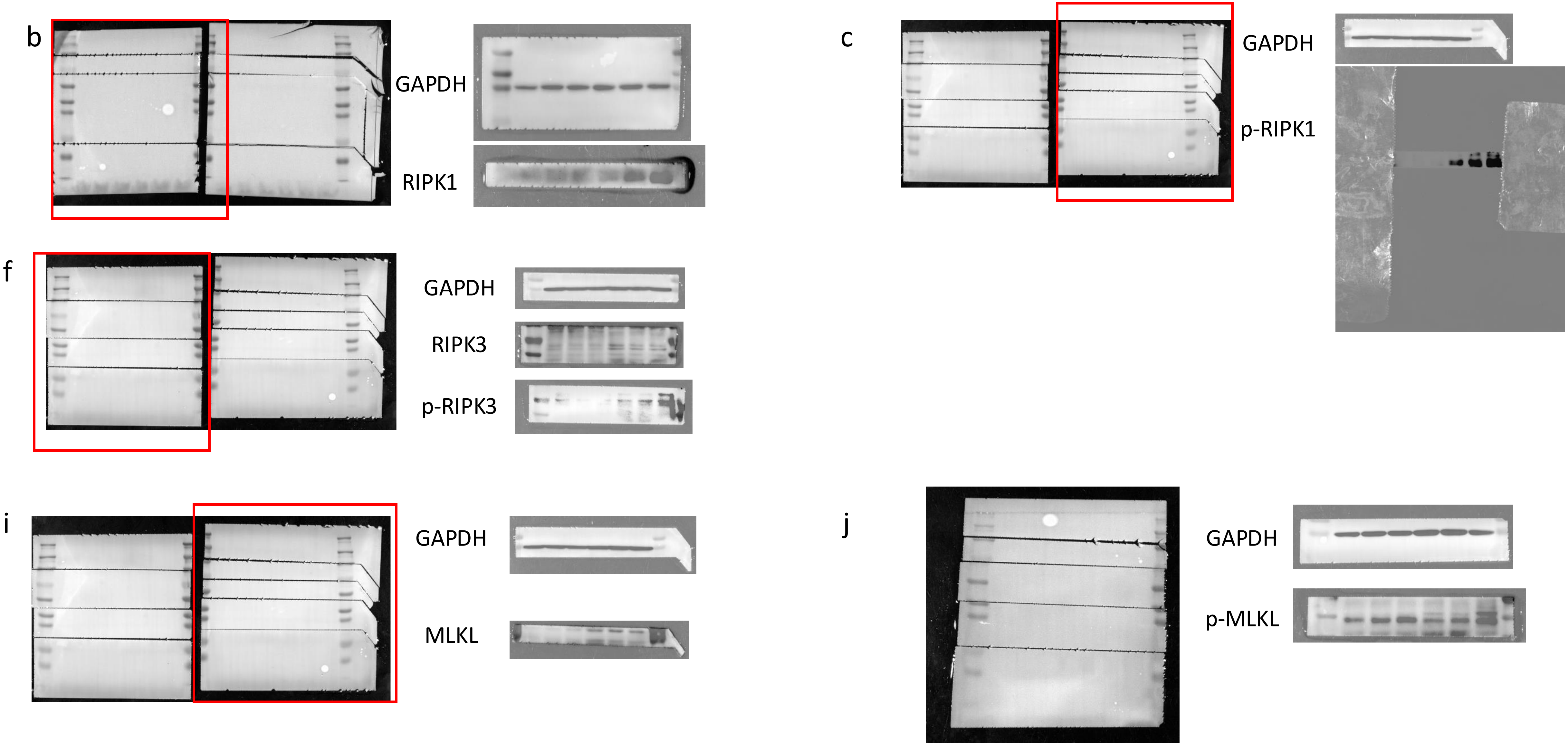
b
GAPDH
RIPK1
c
GAPDH
p-RIPK1
f
GAPDH
RIPK3
p-RIPK3
i
GAPDH
MLKL
j
GAPDH
p-MLKL

Figure S8

c

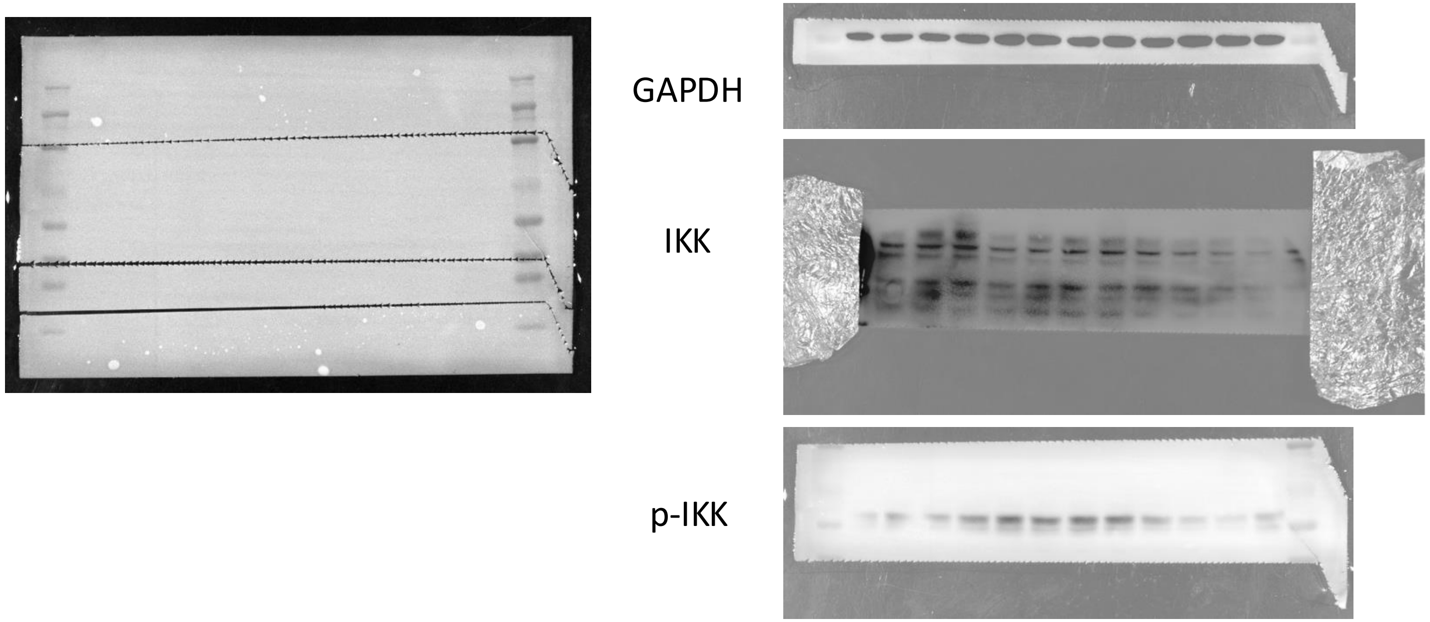

d

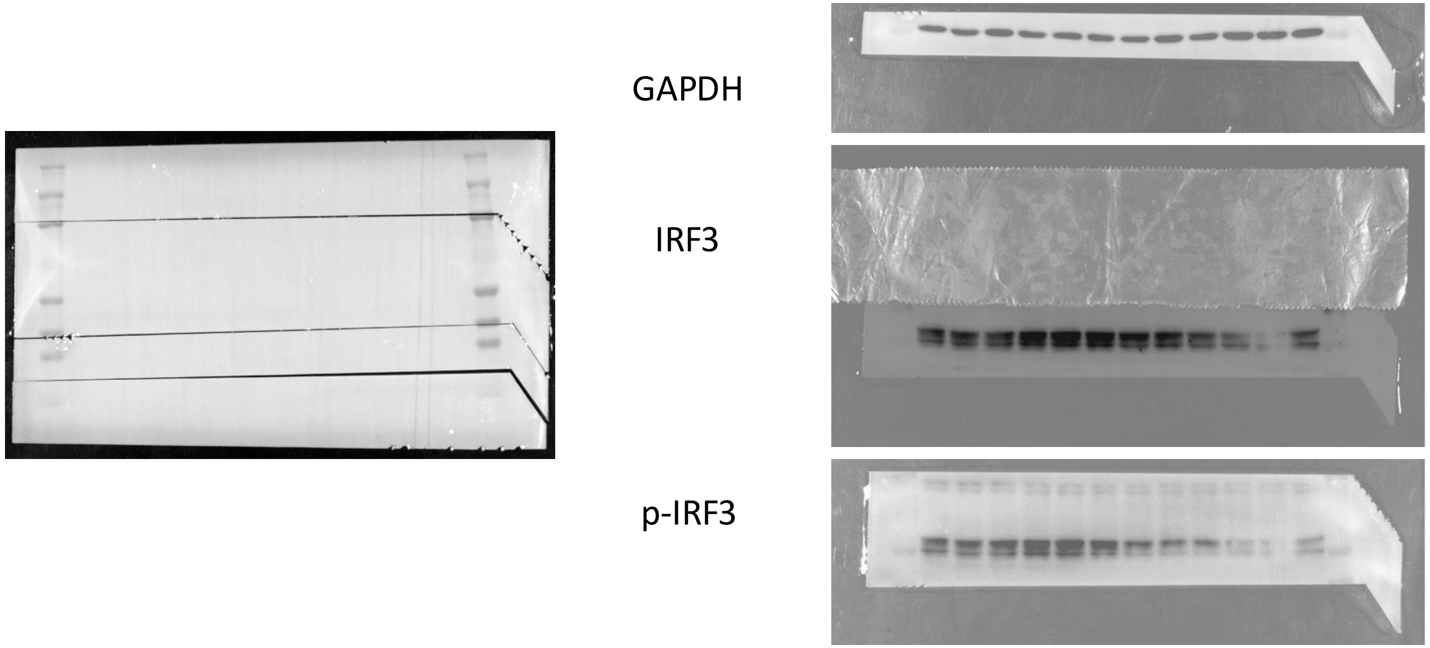

Supplement: Supplementary file 2 — Supplementary Information [file 42003_2025_8010_MOESM2_ESM.pdf]
