# Supplementary material for: Targeting alveolar epithelial cells with lipid micelle-encapsulated necroptosis inhibitors to alleviate acute lung injury
Source: Commun Biol. 2025 Apr 6;8:573. doi: 10.1038/s42003-025-08010-1 (PMC11972349; doi:10.1038/s42003-025-08010-1)
Supplement: Supplementary file 5 — Reporting summary [file 42003_2025_8010_MOESM5_ESM.pdf]

nature portfolio

Corresponding author(s): *Double-anonymous peer review submissions: write DAPR and your manuscript number here instead of author names.*

Last updated by author(s): Feb 21, 2025

Reporting Summary

Nature Portfolio wishes to improve the reproducibility of the work that we publish. This form provides structure for consistency and transparency in reporting. For further information on Nature Portfolio policies, see our Editorial Policies and the Editorial Policy Checklist.

Statistics

For all statistical analyses, confirm that the following items are present in the figure legend, table legend, main text, or Methods section.

n/a	Confirmed	
☐	☒	The exact sample size (*n*) for each experimental group/condition, given as a discrete number and unit of measurement
☐	☒	A statement on whether measurements were taken from distinct samples or whether the same sample was measured repeatedly
☐	☒	The statistical test(s) used AND whether they are one- or two-sided<br>*Only common tests should be described solely by name; describe more complex techniques in the Methods section.*
☐	☒	A description of all covariates tested
☒	☐	A description of any assumptions or corrections, such as tests of normality and adjustment for multiple comparisons
☐	☒	A full description of the statistical parameters including central tendency (e.g. means) or other basic estimates (e.g. regression coefficient) AND variation (e.g. standard deviation) or associated estimates of uncertainty (e.g. confidence intervals)
☐	☒	For null hypothesis testing, the test statistic (e.g. *F*, *t*, *r*) with confidence intervals, effect sizes, degrees of freedom and *P* value noted<br>*Give P values as exact values whenever suitable.*
☒	☐	For Bayesian analysis, information on the choice of priors and Markov chain Monte Carlo settings
☒	☐	For hierarchical and complex designs, identification of the appropriate level for tests and full reporting of outcomes
☐	☒	Estimates of effect sizes (e.g. Cohen's *d*, Pearson's *r*), indicating how they were calculated

Our web collection on statistics for biologists contains articles on many of the points above.

Software and code

Policy information about availability of computer code

Data collection	The RNA sequencing data used in this study are uploaded to GEO-NCBI, with the accession code GSE263867.
Data analysis	The data analysis software for RNA sequencing is R 4.3.1 and RStudio.

For manuscripts utilizing custom algorithms or software that are central to the research but not yet described in published literature, software must be made available to editors and reviewers. We strongly encourage code deposition in a community repository (e.g. GitHub). See the Nature Portfolio guidelines for submitting code & software for further information.

Data

Policy information about availability of data

All manuscripts must include a data availability statement. This statement should provide the following information, where applicable:

- Accession codes, unique identifiers, or web links for publicly available datasets
- A description of any restrictions on data availability
- For clinical datasets or third party data, please ensure that the statement adheres to our policy

The data that support the findings of this study are available from the corresponding author upon reasonable request.

Research involving human participants, their data, or biological material

Policy information about studies with human participants or human data. See also policy information about sex, gender (identity/presentation), and sexual orientation and race, ethnicity and racism.

Reporting on sex and gender	N/A
Reporting on race, ethnicity, or other socially relevant groupings	N/A
Population characteristics	N/A
Recruitment	N/A
Ethics oversight	N/A

Note that full information on the approval of the study protocol must also be provided in the manuscript.

Field-specific reporting

Please select the one below that is the best fit for your research. If you are not sure, read the appropriate sections before making your selection.

☒ Life sciences ☐ Behavioural & social sciences ☐ Ecological, evolutionary & environmental sciences

For a reference copy of the document with all sections, see nature.com/documents/nr-reporting-summary-flat.pdf

Life sciences study design

All studies must disclose on these points even when the disclosure is negative.

Sample size	The cell experiments in this study were performed with 3-5 samples per group. If a statistically significant difference is obtained with a small sample size, no further sample size increase is made. In animal experiments, each group consists of 5-7 samples, considering individual differences among animals and potential factors such as transportation and housing that may affect the animals' physical and mental status, which could, in turn, influence the experimental results.
Data exclusions	No data were excluded in this study.
Replication	In this study, all cell experiments were repeated 3-5 times, and animal experiments were repeated 3 times.
Randomization	In the experiments, both cell and animal groups were assigned randomly.
Blinding	The data analysis and statistical personnel were not aware of the experimental groupings.

Reporting for specific materials, systems and methods

We require information from authors about some types of materials, experimental systems and methods used in many studies. Here, indicate whether each material, system or method listed is relevant to your study. If you are not sure if a list item applies to your research, read the appropriate section before selecting a response.

Materials & experimental systems

n/a	Involved in the study	
☐	☒	Antibodies
☐	☒	Eukaryotic cell lines
☐	☐	Palaeontology and archaeology
☐	☒	Animals and other organisms
☐	☐	Clinical data
☐	☐	Dual use research of concern
☐	☐	Plants

Methods

n/a	Involved in the study	
☒	☐	ChIP-seq
☒	☐	Flow cytometry
☒	☐	MRI-based neuroimaging

Antibodies

Antibodies used	SPC Antibody (Affinity, #DF6647); RIPK1 (CST, #3493); p-RIPK1 (Affinity, af2398); RIPK3 (Abcam, ab62344) ; p-RIPK3 (Abcam,

Antibodies used

ab209384); MLKL (Merck, SAB5700808); p-MLKL (Abcam, ab196436 and Affinity, AF7420); Caspase-8 (Proteintech, 13423-1-AP) Caspase-3 (Abcam,ab184787); Caspase-7 (Abcam,ab255818)； Affinipure Goat Anti-Mouse IgG (H+L) (Proteintech, SA00001-1); HRP-conjugated Affinipure Goat Anti-Rabbit IgG (H+L) (Proteintech, SA00001-2); β-Actin (Sigma, A2228); GAPDH (Abcam, ab8245) ; anti-E-cadherin (Proteintech, 20874-1-AP) ; Goat Anti-rabbit IgG (HRP) (Abcam, ab205718); Goat Anti-rabbit IgG(HRP) (Abcam, ab205718); Tubulin (Abcam, ab6160, 1:1000); Multi-rAb CoraLite® Plus 594-Goat Anti-Rabbit Recombinant Secondary Antibody (H+L) (Proteintech, RGAR004); Goat anti-Rat IgG (H+L) Cross-Adsorbed Secondary Antibody, Alexa Fluor™ 594 (Thermofisher, A-11007) ; Goat anti-Rabbit IgG (H+L) Cross-Adsorbed Secondary Antibody, Alexa Fluor™ 488 (Thermofisher, A-11008)

Validation

SPC Antibody (Rabbit, ICC, IF, IHC, WB); RIPK1 (Rabbit, WB, IP, IF, Flow Cyt); p-RIPK1 (Rabbit, WB, IHC, IF/ICC); RIPK3 (Rabbit, WB, IHC) ; p-RIPK3 (Rabbit, elisa, Dot blot, WB); MLKL (Rabbit, IF, IHC, WB); p-MLKL (Mouse, WB, IP, Dot blot and Rabbit, WB, IHC); Caspase-8 (Rabbit, WB, IHC, IF/ICC, IP, ELISA); Caspase-3 (Mouse, WB, IHC-P, IP); Caspase-7 (Mouse, Flow Cyt, WB, IHC-P)； Affinipure Goat Anti-Mouse IgG (H+L) (Goat, ELISA, WB, Dot blot); HRP-conjugated Affinipure Goat Anti-Rabbit IgG (H+L) (Goat, ELISA, WB, Dot blot); β-Actin (Mouse, western blot, two-dimensional gel immunoblot, immunocytochemistry); GAPDH (Mouse, WB, ICC/IF) ; anti-E-cadherin (Rabbit, WB, IHC, IF/ICC, IF-P, IF-Fro, IP, CoIP, ELISA, Cell treatment) ; Goat Anti-rabbit IgG (HRP) (Goat, IHC-P, WB, ELISA, IP); Tubulin (Rat, WB, ICC/IF, IHC-P, Flow Cyt); Multi-rAb CoraLite® Plus 594-Goat Anti-Rabbit Recombinant Secondary Antibody (H+L) (Goat, IF, FC); Goat anti-Rat IgG (H+L) Cross-Adsorbed Secondary Antibody, Alexa Fluor™ 594 (Goat, ICC/IF, FC) ; Goat anti-Rabbit IgG (H+L) Cross-Adsorbed Secondary Antibody, Alexa Fluor™ 488 (Rabbit, IHC, ICC/IF, FC)

Eukaryotic cell lines

Policy information about cell lines and Sex and Gender in Research

Cell line source(s)

The human bronchial epithelial cell line (HBE) and mouse alveolar epithelial cell line (MLE) were purchased from American Type Culture Collection (ATCC, CRL-2741, CRL-2110). The source of the cells has been stated in the original text.

Authentication

The cell lines have been authenticated.

Mycoplasma contamination

The cell lines used in this study were not found to be contaminated with mycoplasma.

Commonly misidentified lines
(See ICLAC register)

NA

Palaeontology and Archaeology

Specimen provenance

Male C57BL/6 mice, aged 6-8 weeks, weighing between 16-24 grams, were purchased from SHANGHAI SLAC LABORATORY ANIMAL CO.LTD., and were acclimated for one week in the animal laboratory of the Clinical Research Center of the Second Affiliated Hospital of Zhejiang University School of Medicine. The mice were housed under specific pathogen-free (SPF) conditions, with a temperature of 25 ± 1℃, humidity of 60 ± 5%, a 12 hour light/dark cycle, and free access to water. All animal experiments were approved by the Ethics Review Committee of the Second Affiliated Hospital of Zhejiang University School of Medicine (No. 2022.050).

Specimen deposition

The specimens are stored at the Center for Basic and Translational Research, Second Affiliated Hospital, Zhejiang University School of Medicine.

Dating methods

The specimen of this study are available from the corresponding author upon reasonable request.

☒ Tick this box to confirm that the raw and calibrated dates are available in the paper or in Supplementary Information.

Ethics oversight

All animal experiments were approved by the Ethics Review Committee of the Second Affiliated Hospital of Zhejiang University School of Medicine (No. 2022.050).

Note that full information on the approval of the study protocol must also be provided in the manuscript.

Animals and other research organisms

Policy information about studies involving animals; ARRIVE guidelines recommended for reporting animal research, and Sex and Gender in Research

Laboratory animals

Male C57BL/6 mice, aged 6-8 weeks, weighing between 16-24 grams, were purchased from SHANGHAI SLAC LABORATORY ANIMAL CO.LTD., and were acclimated for one week in the animal laboratory of the Clinical Research Center of the Second Affiliated Hospital of Zhejiang University School of Medicine.

Wild animals

NA

Reporting on sex

The results of this study are applicable to all natural sexes.

Field-collected samples

The mice were housed under specific pathogen-free (SPF) conditions, with a temperature of 25 ± 1℃, humidity of 60 ± 5%, a 12 hour light/dark cycle, and free access to water.

Ethics oversight

All animal experiments were approved by the Ethics Review Committee of the Second Affiliated Hospital of Zhejiang University School of Medicine (No. 2022.050).

Note that full information on the approval of the study protocol must also be provided in the manuscript.

Clinical data

Policy information about clinical studies

All manuscripts should comply with the ICMJE guidelines for publication of clinical research and a completed CONSORT checklist must be included with all submissions.

Clinical trial registration	NA
Study protocol	NA
Data collection	NA
Outcomes	NA

Dual use research of concern

Policy information about dual use research of concern

Hazards

Could the accidental, deliberate or reckless misuse of agents or technologies generated in the work, or the application of information presented in the manuscript, pose a threat to:

No	Yes	
☒	☐	Public health
☒	☐	National security
☒	☐	Crops and/or livestock
☒	☐	Ecosystems
☒	☐	Any other significant area

Experiments of concern

Does the work involve any of these experiments of concern:

No	Yes	
☒	☐	Demonstrate how to render a vaccine ineffective
☒	☐	Confer resistance to therapeutically useful antibiotics or antiviral agents
☒	☐	Enhance the virulence of a pathogen or render a nonpathogen virulent
☒	☐	Increase transmissibility of a pathogen
☒	☐	Alter the host range of a pathogen
☒	☐	Enable evasion of diagnostic/detection modalities
☒	☐	Enable the weaponization of a biological agent or toxin
☒	☐	Any other potentially harmful combination of experiments and agents

Plants

Seed stocks	NA
Novel plant genotypes	NA
Authentication	NA
